# Influence of Cellular Aging on Liver Stiffness in Patients With Hepatitis C Virus Achieving Sustained Viral Response

**DOI:** 10.1093/infdis/jiaf087

**Published:** 2025-02-19

**Authors:** Alejandro Gonzalez-Serna, Anaïs Corma-Gomez, Mercedes Cano, Ricardo Rubio-Sánchez, Carmen Martín-Sierra, Pilar Rincón, Jesica Martín-Carmona, Margarita Pérez, Juan Antonio Pineda, Luis Miguel Real, Juan Macias

**Affiliations:** Grupo Virología e ITS, Hospital Universitario Virgen de Valme, Sevilla, Spain; Facultad de Farmacia, Departamento de Fisiología, Universidad de Sevilla, Sevilla, Spain; Instituto de Biomedicina de Sevilla, Sevilla, Spain; Centro de Investigación Biomédica en Red de Enfermedades Infecciosas, Spain; Grupo Virología e ITS, Hospital Universitario Virgen de Valme, Sevilla, Spain; Instituto de Biomedicina de Sevilla, Sevilla, Spain; Centro de Investigación Biomédica en Red de Enfermedades Infecciosas, Spain; Facultad de Farmacia, Departamento de Fisiología, Universidad de Sevilla, Sevilla, Spain; Servicio de Análisis Clínicos, Hospital Universitario Virgen de Valme, Sevilla, Spain; Grupo Virología e ITS, Hospital Universitario Virgen de Valme, Sevilla, Spain; Instituto de Biomedicina de Sevilla, Sevilla, Spain; Centro de Investigación Biomédica en Red de Enfermedades Infecciosas, Spain; Grupo Virología e ITS, Hospital Universitario Virgen de Valme, Sevilla, Spain; Grupo Virología e ITS, Hospital Universitario Virgen de Valme, Sevilla, Spain; Grupo Virología e ITS, Hospital Universitario Virgen de Valme, Sevilla, Spain; Grupo Virología e ITS, Hospital Universitario Virgen de Valme, Sevilla, Spain; Departamento de Medicina, Universidad de Sevilla, Sevilla, Spain; Grupo Virología e ITS, Hospital Universitario Virgen de Valme, Sevilla, Spain; Instituto de Biomedicina de Sevilla, Sevilla, Spain; Centro de Investigación Biomédica en Red de Enfermedades Infecciosas, Spain; Departamento de Bioquímica Médica, Biología Molecular e Inmunología, Universidad de Sevilla, Sevilla, Spain; Grupo Virología e ITS, Hospital Universitario Virgen de Valme, Sevilla, Spain; Instituto de Biomedicina de Sevilla, Sevilla, Spain; Centro de Investigación Biomédica en Red de Enfermedades Infecciosas, Spain; Departamento de Medicina, Universidad de Sevilla, Sevilla, Spain

**Keywords:** cellular aging, liver stiffness, telomere length, SVR, DAA

## Abstract

**Background:**

Liver stiffness (LS) is not reduced in 10%–30% of patients who achieve sustained viral response (SVR) after hepatitis C virus (HCV) elimination with direct-acting antivirals (DAA). Our aim was to analyze whether the parameters associated with cellular aging measured at the DAA initiation date are related to LS reduction upon achieving SVR.

**Methods:**

In a prospective cohort study (GEHEP-011) we measured several parameters associated with cellular aging, such as telomere attrition, mitochondrial alterations, and soluble biomarkers associated with senescence-associated secretory phenotype at the DAA initiation date, and examined their associations with a significant (≥20%) LS decrease at the SVR time point.

**Results:**

In total, 175 individuals were included in this study. In 101 (57.7%) patients, the LS reduction was ≥20% at SVR. In the multivariate analysis adjusted for sex, age, CXCL10, hsPCR, and CCL11 levels, greater relative telomere length (RTL) emerged as the sole variable independently associated with a significant LS decrease in SVR (1.102; 95% confidence interval, 1.001–1.1214; *P* = .047). Furthermore, changes in LS, including significant decrease, decrease <20%, or increase, were congruently associated with RTL (*P* = .011).

**Conclusions:**

Greater RTL was independently associated with a significant LS reduction in SVR. Thus, increased cellular aging may be responsible for the absence of liver regeneration after HCV eradication. Further studies are required to assess the long-term effects of cellular aging after SVR.

**Clinical Trials Registration:**

NCT04460157.

## Teaser

Liver stiffness is not reduced in 10%–30% of patients who achieve sustained viral response (SVR) after HCV elimination. Here, we report that greater relative telomere size is independently associated with a significant liver stiffness reduction in SVR.

## Graphical Abstract

**Graphical Abstract:**
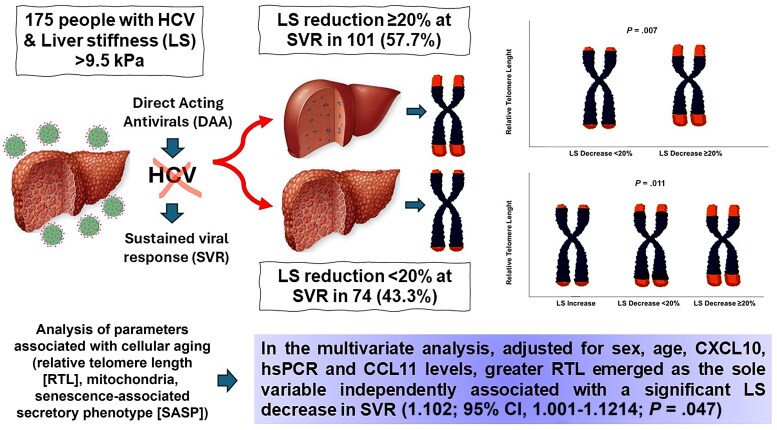

The cure of hepatitis C virus (HCV) infection with direct-acting antiviral (DAA)-based therapy is associated with a reduction in liver injury in most patients, and therefore has a lower risk of clinical events. Achieving a sustained viral response (SVR) with DAA-based therapy is associated with a dramatic decrease in the risk of liver complications in people eliminating HCV [1, 2]. However, some liver complications may occur even in the long-term [3, 4]. In this regard, a reduced liver stiffness (LS) measured by vibration-controlled transient elastography at SVR is a marker of subsequent hepatic outcome in people who achieve SVR after treatment with DAAs [5, 6]. However, in 10%–30% of patients, LS does not decline after achieving SVR with DAAs [7, 8]. The enduring presence of liver injury may influence both overall mortality and mortality attributable to other etiologies in patients who have achieved a cure for active HCV infection [9]. The mechanisms responsible for this lack of LS reduction after HCV elimination with DAA are not completely understood.

Cellular aging is involved in a large number of pathologies and their evolution, such as liver disease, and could impact the evolution of patients after SVR. Numerous parameters linked to cellular aging have been identified [10], and within this set, certain factors may be of particular significance in the progression of liver disease. These include antiretroviral treatment [8], mitochondrial damage [11], telomere length [12], as well as various indicators of chronic systemic immune activation [13], inflammation [14], and senescence [15], encompassing the senescence-associated secretory phenotype [16]. Other nutritional parameters [17] are noteworthy. Moreover, because they share transmission routes, a high percentage of individuals with HCV also live with human immunodeficiency virus (HIV), and both HIV and antiretroviral therapy (ART) can aggravate several parameters related to cellular aging in these individuals [8, 10].

As mentioned above, the persistence of hepatic damage in patients who achieve SVR entails a high risk of comorbidities as well as the need for close monitoring. Therefore, it is necessary to conduct studies to analyze the relationship between changes in LS after SVR and cellular aging to better understand the cellular mechanisms underlying this complex process. Consequently, our aim was to analyze the parameters associated with cellular aging just before the administration of DAA and to determine if there is an association with the reduction in LS upon achieving SVR.

## METHODS

### Patients and Study Design

Patients included in the prospective GEHEP-011 (clinicaltrials.gov NCT04460157) cohort, evaluated in 1 of the participating centers in Southern Spain from October 2011 to October 2023, were recruited in this prospective study. Patients who fulfilled the following criteria were included: (1) achievement of SVR 12 weeks after the end of treatment with interferon-free DAA regimens; (2) LS ≥ 9.5 kPa measured within 3 months prior to DAA initiation; and (3) availability of a LS measurement at the SVR time point and a serum sample at the DAA initiation date. Subjects seropositive for HBsAg were excluded from this study. All participants were evaluated using a common protocol. LS was assessed using vibration-controlled transient elastography (FibroScan; Echosens), according to a standardized procedure [18], within 30 days before starting DAA therapy and on the day of SVR. An experienced operator performed all examinations at each participating institution. M probe was used for all patients. Determinations were considered evaluable if they included at least 10 measurements, with a success rate of ≥60% and an interquartile range of <30% of the median. According to previous studies conducted in people with HIV (PWH)/HCV, individuals with baseline LS >14 kPa were considered to have cirrhosis [8].

### Laboratory Determinations

#### Telomere Attrition and Mitochondrial Copy Number: Parameters of Senescence

Cell-free DNA (cfDNA) was isolated from serum samples using the MagMAX Cell-Free Total Nucleic Acid Isolation Kit (Thermo Fisher Scientific) according to the manufacturer's specifications. The DNA concentration of each sample was evaluated by spectrophotometry using Nanodrop (Thermo Scientific) according to the manufacturer's specifications. Relative telomere length (RTL) was measured in cfDNA using the Absolute Human Telomere Length Quantification quantitative polymerase chain reaction (qPCR) Assay Kit (AHTLQ; ScienCell Research Laboratories) according to the manufacturer's instructions. The mitochondrial copy number was measured in cfDNA using the Absolute Human Mitochondrial DNA Copy Number Quantification qPCR Assay Kit (AHMQ; ScienCell Research Laboratories) according to the manufacturer's instructions.

#### Soluble Biomarkers Associated With Senescence-Associated Secretory Phenotype

The following markers were assessed: parameters of inflammation were high-sensitivity C-reactive protein (hsCRP; Roche latex-enhanced immunoturbidimetric assay), interleukin 6 (IL-6; Diaclone), chemokine C-X-X ligand with motif 10 (CXCL10; Invitrogen), chemokine C-C ligand with motif 11 (CCL11; Sigma-Aldrich), and chitinase-3-like protein 1 (CHI3L1; Invitrogen); activation parameters were soluble CD14 (sCD14; FineTest) and soluble CD163 (sCD163; FineTest); oxidative stress parameter was isoprostanate-8 (Iso-8; Cayman Chemical); hemostasis parameter was von Willebrand Factor (vWF; Invitrogen); and parameter of physiological function was vitamin D (MyBioSource).

### Statistical Analyses

The main outcome variable was a clinically significant decrease (≥20%) in LS from the initiation of DAA to SVR, which is associated with a significant reduction in the risk of patient decompensation or liver-related death, in accordance with recent studies [19–21]. Additionally, we divided our population into 3 groups based on whether they had a reduction in LS of ≥20% in SVR, a reduction of <20% in SVR, or an increase in LS from the initiation of DAA until SVR, and analyzed the characteristics of each group in relation to the parameters included in the study. Values were compared using the Mann-Whitney test or Wilcoxon test, the Kruskal-Wallis test was used for the comparison of the 3 groups, and the χ^2^ test was used to compare proportions. Variables associated with a significant LS decrease from DAA initiation to the date of SVR evaluation in the univariate analysis with a *P* value ≤.1, along with age and sex, were entered into the multivariate logistic regression model. Greater accelerated aging has been reported in individuals who acquire HIV than in those who acquire HCV [12]. Therefore, we conducted a sensitivity analysis restricted to PWH. In addition, treatment with Nonnucleoside reverse transcriptase inhibitors (NNRTI) plus 2 nucleoside reverse transcriptase inhibitors (NRTIs) has been associated with a greater LS decline than other ART combinations in PWH/HCV achieving SVR with DAA-based therapy [8]. For this reason, we divided PWH into 2 groups: these were (1) a group receiving NNRTI plus 2 NRTI-based ART, and (2) a group receiving other ART combinations. All data analyses were performed using SPSS statistical software package release 27.0 (IBM).

### Ethical Aspects

Both the study design and development complied with the Helsinki and Istanbul Declarations and were approved by the local ethics committee of the Hospital Universitario Virgen de Valme (Seville) (reference No. 0031-N-19). All the patients provided written informed consent when they entered the cohort.

## RESULTS

### Characteristics of the Patients

In total, 175 patients were enrolled in this study. Most of them were male, had low alcohol consumption, and approximately half of them were PWH (Table 1). LS values transitioned from a median of 16.3 kPa (Q1–Q3, 11.8–28 kPa) at DAA initiation to 12.3 kPa (Q1–Q3, 8.2–21.3 kPa) at SVR (*P* < .001). The difference in LS between the start of DAA and SVR was −4.6 kPa (Q1–Q3, −8.7 to −1 kPa), representing a percentage reduction of −27.2% (Q1–Q3, −45.1% to −5.3%).

**Table 1.** Characteristics of the Patients at Direct-Acting Antivirals Initiation

Variable	Value (n = 175)
Male sex, n (%)	147 (84)
Age, y, median (Q1–Q3)	51.4 (48.1–54.7)
PWID, n (%)	128 (73.1)
Alcohol <50 g/d, n (%)^a^	165 (95.9)
Coinfection HCV/HIV, n (%)	98 (56)
Cirrhosis, n (%)	102 (58.3)
CPT class A, n (%)	166 (94.9)
MELD index ≥10, n (%)	24 (13.9)

Abbreviations: CPT, Child-Pugh-Turcotte; HCV, hepatitis C virus; HIV, human immunodeficiency virus; MELD, model for end-stage liver disease; PWID, people with current or past injecting drug use.

^a^In the last 6 months. Individuals showing baseline liver stiffness >14 kPa were considered cirrhotic.

### Significant LS Decrease in SVR

Among the 175 patients included in the study, 101 (57.7%) experienced a significant LS decrease in SVR, with the LS values transitioning from a median of 16.7 kPa (Q1–Q3, 11.9–28.6 kPa) at DAA initiation to 11.6 kPa (Q1–Q3, 7.6–18.4 kPa) at SVR. The difference in LS between the start of DAA and SVR was −7.2 kPa (Q1–Q3, −13.2 to −4.9 kPa), which represented a percentage reduction of −42.6% (Q1–Q3, −51.5% to −32.8%). On the other hand, 74 (42.3%) patients did not experience a significant LS decrease in SVR, with the LS values transitioning from a median of 14.6 kPa (Q1–Q3, 11.4 to 27.5 kPa) at DAA initiation to 17 kPa (Q1–Q3, 11.9 to 30.3 kPa) at SVR (*P* = .414). The difference in LS between the start of DAA and SVR was −0.6 kPa (Q1–Q3, −1.6 to 3.3 kPa), which represented a percentage reduction of −2.9% (Q1–Q3, −10.6% to 20.8%).

**Figure 1.**
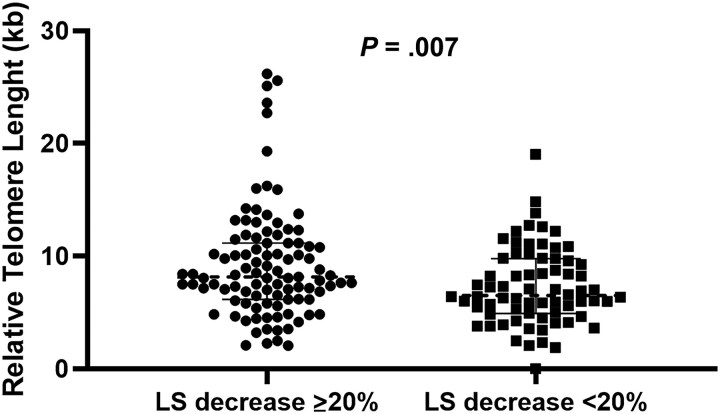
Relative telomere length between the 2 groups. Abbreviation: LS, liver stiffness.

There were no statistically significant differences at the initiation of DAA between the group of patients with significant LS decrease and the group with no significant LS decrease in the following variables: age, intravenous drug use, alcohol consumption, HIV coinfection, cirrhosis, Child-Pugh-Turcotte class A, model for end-stage liver disease (MELD) index, or LS levels before administration of DAAs (Table 2). There was a higher proportion of women in the group of patients who experienced a significant decrease in LS (Table 2). When we analyzed the association between a significant LS decrease in SVR and the parameters related to cellular aging included in the study, univariate analysis showed that the parameters of senescence RTL and the inflammation marker CXCL10 were associated with a significant LS decrease in SVR. In the multivariate analysis adjusted for sex, age, CXCL10, hsPCR, and CCL11 levels, greater RTL emerged as the sole variable independently associated with a significant decrease in LS in SVR (1.102; 95% confidence interval [CI], 1.001–1.1214; *P* = .047; Table 2). The significant differences in RTLs between the 2 groups are shown in Figure 1.

**Table 2.** Variables Associated With a Significant (≥20%) LS Decrease in SVR

Variable (n = 175)	Significant LS Decrease (n = 101)	No Significant LS Decrease (n = 74)	*P* Value	Adjusted Odds Ratio (95% CI)	*P* Value
Male sex, n (%)	79 (78.2)	68 (91.9)	.021	0.349 (.103–1.178)	.090
Age, y	51.8 (47.7–54.8)	50.5 (48.3–54.5)	.840	0.981 (.941–1.024)	.388
PWID, n (%)	69 (68.3)	59 (79.7)	.120		
Alcohol <50 g/d, n (%)^a^	93 (94.9)	72 (97.3)	.700		
Coinfection HCV/HIV, n (%)	52 (51.5)	46 (62.2)	.169		
Cirrhosis, n (%)	63 (62.4)	39 (52.7)	.217		
CPT class A, n (%)	95 (94.1)	71 (95.9)	1.000		
MELD index ≥10, n (%)	12 (12.1)	12 (16.2)	.508		
LS pre-DAA, KPa	17.1 (12–29.2)	14.5 (14.4–27.5)	.126		
Senescence biomarkers					
RTL, kb	8.17 (6.15–11.16)	6.5 (4.91–9.79)	.007	1.102 (1.001–1.214)	.047
mtDNA-CN	149 (84–271)	144 (94–200)	.444		
Inflammation biomarkers					
hsPCR, mg/L	0.77 (0.34–1.99)	1.08 (0.52–3.13)	.056	0.969 (.899–1.045)	.417
IL-6, pg/mL	1.47 (0.66–2.58)	1.79 (0.76–3.29)	.190		
CXCL10, pg/mL	99.3 (95.2–189.9)	95.9 (94.5–174.1)	.027	1.006 (.998–1.014)	.170
CCL11, pg/mL	390 (277–505)	470 (315–557)	.093	1.000 (.998–1.001)	.598
CHI3L1, pg/mL	782 (416–1185)	859 (351–1399)	.349		
Activation biomarkers					
sCD163, ng/mL	49.2 (39.8–58.9)	46.9 (39.7–55.1)	.276		
sCD14, ng/mL	0.73 (0.6–0.98)	0.76 (0.63–1.02)	.422		
Hemostasis biomarker					
vWF, ng/mL	26.8 (16.1–48.8)	27.1 (18–47.8)	.773		
Oxidative stress biomarker					
Iso-8, pg/mL	143 (129–158)	135 (126–160)	.378		
Physiological functions biomarker					
Vitamin D, ng/mL	61.6 (49.3–72.5)	61.8 (47.2–73.4)	.981		

Data are median (Q1–Q3) except where indicated.

Abbreviations: CCL11, chemokine C-C ligand with motif 11; CHI3L1, chitinase-3-like protein 1; CPT, Child-Pugh-Turcotte; CXCL10, chemokine C-X-C ligand with motif 10; DAA, direct-acting antiviral; HCV, hepatitis C virus; HIV, human immunodeficiency virus; hsPCR, high-sensitivity C-reactive protein; IL-6, interleukin 6; Iso-8, isoprostanate-8; LS, liver stiffness; MELD, model for end-stage liver disease; mtDNA-CN, No. of copies of mitochondrial DNA; PWID, people with current or past injecting drug use; RTL, relative telomere length; sCD14, soluble CD14; sCD163, soluble CD163; SVR, sustained viral response; vWF, von Willebrand Factor.

^a^In the last 6 mo; individuals showing baseline LS >14 kPa were considered to have cirrhosis.

**Figure 2.**
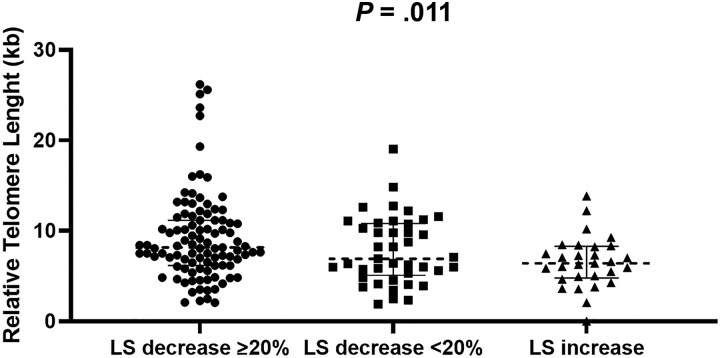
Relative telomere length among the 3 groups. Abbreviation: LS, liver stiffness.

We divided the population into 3 groups based on whether they had a reduction in LS of ≥20% in SVR, a reduction of <20% in SVR, or an increase in LS from the initiation of DAA until SVR. The changes in LS in these groups are summarized in Table 3. Interestingly, although no significant differences were found when comparing the baseline LS of the 3 groups, significant differences in RTL were observed among these groups, transitioning from a smaller size in the group that experienced an increase in LS to the largest size in the group that experienced an LS decrease of ≥20% (Figure 2).

**Table 3.** Comparison of LS Among the 3 Groups From the Initiation of DAA Until SVR

Variable (n = 175)	LS Decrease ≥ 20% (n = 101)	LS Decrease < 20% (n = 43)	LS Increase (n = 31)	*P* Value^a^
LS pre-DAA, KPa	16.7 (11.9 to 28.6)	14.6 (11.5 to 27)	14.4 (10.3 to 27.7)	.298
LS at SVR, KPa	11.6 (7.6 to 18.4)	13.1 (10.1 to 26)	21.3 (14.4 to 46.4)	<.001
*P* value^b^	<.001	<.001	<.001	
LS change, KPa	−7.2 (−13.2 to −4.9)	−1.5 (−2.5 to −0.7)	4.2 (1.8 to 9.3)	<.001
LS change, %	−42.6 (−51.5 to −32.8)	−9.7 [−13.8 to −5.1]	22.2 (10.2 to 64.7)	<.001

Data are median (Q1 to Q3).

Abbreviations: DAA, direct-acting antiviral; LS, liver stiffness; SVR, sustained viral response.

^a^Analysis of the 3 groups using the Kruskal-Wallis test.

^b^Analysis of LS change from the start of DAA to SVR using Wilcoxon test.

### Comparison of PWH and People Without HIV

Comparing PWH with people without HIV, we observed that in 52 out of 98 cases (53.1%) of PWH, there was a decrease in LS ≥20% in SVR, whereas this significant decrease in LS occurred in 49 out of 77 cases (63.6%) in people without HIV (*P* = .160). In PWH, LS values decreased from a median of 16.6 kPa (Q1–Q3, 11.9–29.3 kPa) at DAA initiation to 12.2 kPa (Q1–Q3, 8.8–21.9 kPa) at SVR (*P* < .001). On the other hand, LS values changed from a median of 16.1 kPa (Q1–Q3, 11.7–27.4 kPa) at DAA initiation to 13.1 kPa (Q1–Q3, 7.2–21 kPa) at SVR in people without HIV (*P* < .001). In PWH, the change in LS between the start of DAA and SVR was −3.9 kPa (Q1–Q3, −7.8 to −0.6 kPa), compared to −5.2 kPa (Q1–Q3, −10.3 to −1.5 kPa) in people without HIV (*P* = .144). In PWH, LS reduction was −23.1% (Q1–Q3, −42.9% to −3.5%), compared to −35.2% (Q1–Q3, −46.1% to −9.1%) in people without HIV (*P* = .167). The RTL in PWH was 7.52 kb (Q1–Q3, 5.86–10.8 kb), while in people without HIV it was 7.58 kb (Q1–Q3, 4.82–10.06 kb) (*P* = .367). The mean age of PWH was 51 years (Q1–Q3, 48–54 years), while that of people without HIV was 52 years (Q1–Q3, 47–56 years) (*P* = .540). We did not find significant differences in any of the analyzed parameters, except for CXCL11, which showed values in PWH of 479.6 ng/mL (Q1–Q3, 316.3–568.7 ng/mL), compared to 367.4 pg/mL (Q1–Q3, 275.5–475.5 pg/mL) in people without HIV (*P* = .002).

### ART Influence in HIV

A comparison of 23 (23.5%) PWH on NNRTI plus 2 NRTIs versus 75 (76.5%) on other ART combinations showed that in 15 out of 23 cases (65.2%) in the NNRTI plus 2 NRTIs group, there was a decrease in LS ≥20% at SVR, whereas this significant decrease in LS occurred in 37 out of 75 cases (49.3%) in the other ART combination groups (*P* = .182). In the NNRTI plus 2 NRTIs group, LS values transitioned from a median of 17.8 kPa (Q1–Q3, 13.8–28 kPa) at DAA initiation to 11.7 kPa (Q1–Q3, 8.3–18.5 kPa) at SVR (*P* < .001). On the other hand, LS values transitioned from a median of 15.7 kPa (Q1–Q3, 11.8–29.9 kPa) at DAA initiation to 13.1 kPa (Q1–Q3, 8.8–22 kPa) at SVR in the other ART combination groups (*P* < .001). In the NNRTI plus 2 NRTIs group, the change in LS between the start of DAA and SVR was −6.6 kPa (Q1–Q3, −9.9 to −1.6 kPa), compared to −3.3 kPa (Q1–Q3, −5.8 to 0.3 kPa) in the other ART combinations group (*P* = .059). The percentage LS reduction in the NNRTI plus 2 NRTIs group was −28.2% (Q1–Q3, −50.9% to −14.3%) compared to −16.9% (Q1–Q3, −40.9% to −2.9%) in the other ART combination groups (*P* = .112). The RTL in the NNRTI plus 2 NRTIs group was 8.58 kb (Q1–Q3, 6.23–11.45 kb), while in the other ART combinations group it was 7.47 kb (Q1–Q3, 5.84–10.77 kb) (*P* = .232). No significant differences were found in any of the parameters analyzed.

## DISCUSSION

In this study, we have shown that a longer RTL measured in cfDNA prior to DAA initiation is independently associated with a significant LS reduction in SVR. Furthermore, changes in LS, that is significant decrease, decrease of <20%, or increase, were congruently associated with RTL. This supports our initial hypothesis, which posits that greater cellular aging could be responsible, at least partially, for the absence of an LS decrease in patients who achieve SVR.

Measurement of RTL in cfDNA is known to reflect the length of telomeres in cells [22]. Furthermore, RTL levels in serum have been observed to decrease with age in healthy individuals, confirming that cfDNA is derived from somatic cells in which telomere length decreases with increasing age [23]. cfDNA plays a significant role in cellular senescence and the associated proinflammatory secretome. Thus, telomere shortening, a hallmark of aging, leads to the depletion of telomeric sequences in the cfDNA pool, thereby unleashing their potential to activate the innate immune system in an age-related manner [23]. Shorter telomeres are indicative of cellular aging and have been linked to increased inflammation and hepatocyte senescence [24]. This reduction in RTL in cfDNA may not only be related to increased inflammation and hepatocellular necrosis, which could affect liver regeneration in SVR but could also have important implications for a higher likelihood of developing hepatocarcinoma [25].

In people with HCV, chronic inflammation can accelerate telomere shortening, leading to compromised cellular repair mechanisms and a reduced regenerative capacity of hepatocytes [26]. The persistence of inflammation and immune activation, despite viral clearance, can impede liver recovery and regeneration [27]. This is crucial because LS is a surrogate marker for fibrosis and portal hypertension, which are critical determinants of clinical outcomes [6]. After achieving SVR, a reduction in LS is expected due to decreased hepatic inflammation and fibrosis regression. However, the extent of LS reduction may be limited in individuals with significant preexisting telomere shortening and sustained inflammatory responses [28]. These factors can perpetuate portal hypertension even after viral eradication, impacting clinical outcomes, such as hepatic decompensation and overall survival. In the present study, inflammatory parameters, such as hsCRP and CXCL10, along with female sex, exhibited univariate associations with a significant decline in LS in SVR. However, although some associations have been observed between these inflammatory markers, female sex, and LS [29, 30], in our study, RTL took precedence, emerging as the only associated variable in the multivariate analysis. Accordingly, cellular aging, marked by reduced RTL, adversely affects hepatocyte function and regenerative potential. Therefore, individuals with shorter telomeres might experience less pronounced improvements in LS and remain at risk for liver-related events and reduced survival rates. Moreover, it has been reported that there could be a higher level of cellular aging in individuals with PWH/HCV versus people with HCV [12, 31]. Consequently, one might expect a lower proportion of people to achieve a significant decrease in LS in SVR. However, this was not observed in our study, which is consistent with the findings of previous studies [32]. Finally, a trend towards a greater reduction in liver damage was observed in PWH treated with NNRTI plus 2 NRTIs, which is consistent with previous findings [8]. However, we likely lacked the statistical power to observe significant differences related to ART as data were available for this subanalysis from only half of the individuals included in the study.

Cellular aging involves intricate mechanisms that may adversely affect liver regeneration after SVR. For example, irreversible growth arrest, altered gene expression, and secretion of proinflammatory cytokines may impede hepatic regeneration [33]. Measuring cellular aging markers may be useful in predicting clinical outcomes and identifying patients at a higher risk of complications after HCV elimination, thus facilitating personalized management strategies and improving patient care. Unfortunately, we did not evaluate aging markers after SVR, which is a limitation of the present study.

In conclusion, in this study, we observed that a longer RTL, which is one of the main parameters of cellular aging, influences the achievement of a significant LS reduction in SVR in individuals who eliminate HCV with DAA. Therefore, cellular aging could be related to the absence of hepatic regeneration that some patients experience after HCV clearance. Further studies are required to assess the long-term impact of cellular aging on the evolution of patients after HCV elimination.

## Notes

***Acknowledgments.*** This study would not have been possible without the collaboration of all people with HIV, medical and nursing staff, and data managers who participated in the study.

***Author contributions.*** A. G. S. performed experiments and analyses, and wrote the manuscript. J. M. codesigned the study, collated multicentric clinical data, and supported data interpretation. L. M. summarized clinical data and supported the statistical analyses. M. C. and R. S. R. performed key experiments. A. C. G., P. R., C. M. S., J. M. S., A. C. G., M. P., and J. A. P. provided the clinical data and supervised the work.

***Data and code availability.*** All data used for the analyses in this study are available from the corresponding author upon reasonable request. This code is available from the corresponding author upon request.

***Financial support.*** This work was supported by Instituto de Salud Carlos III (ISCIII) (grant number PI20/01457 to A. G. S.), cofunded by European Regional Development Fund (ERDF), A Way to Make Europe.
